# External fixator combined with three different fixation methods of fibula for treatment of extra-articular open fractures of distal tibia and fibula: a retrospective study

**DOI:** 10.1186/s12891-020-03840-y

**Published:** 2021-01-04

**Authors:** Dong-Dong Sun, Dan Lv, Kun Zhou, Jian Chen, Li-Lan Gao, Ming-Lin Sun

**Affiliations:** 1 Department of Orthopedic, Characteristic Medical center of Chinese People’s Armed Police Force, No. 220 Cheng Lin Road, Tianjin, 300171 China; 2 Logistics University of People’s Armed Police, Tianjin, 300300 China; 3 School of Mechanical Engineering, Tianjin University of Technology, No. 391 Bin Shui West Road, Tianjin, 300384 China

**Keywords:** Tibial fractures, Fibula, Fractures, open, External fixator, Kirschner wire

## Abstract

**Background:**

To compare the efficacy of three different fixation methods of fibula combined with external fixation of tibia for the treatment of extra-articular open fractures of distal tibia and fibula.

**Methods:**

From January 2017 to July 2019, 91 cases of open fractures of distal tibia and fibula were treated with external fixator, and the fibula was fixed with non-fixation (group A, *n* = 35), plate-screw (group B, *n* = 30) and Kirschner wire (group C, *n* = 26). The operation time, intraoperative blood loss, surgical and implants costs, fracture healing time, postoperative complications, and American Orthopaedic Foot and Ankle surgery (AOFAS) scores were compared among the groups.

**Results:**

Four patients were lost to follow-up, and 87 patients were followed up for 5–35 months (average, 14.2 months). The operation time of group C (114.92 ± 36.09 min) was shorter than that of group A (142.27 ± 47.05 min) and group B (184.00 ± 48.56 min) (*P* < 0.05). There was no difference in intraoperative blood loss among the three groups (*P* > 0.05). The surgical and implants costs in group C (5.24 ± 1.21, thousand dollars) is lower than that in group A (6.48 ± 1.11, thousand dollars) and group B (9.37 ± 2.16, thousand dollars) (*P* < 0.05). The fracture healing time of group C (5.67 ± 1.42 months) was significantly less than that of group A (6.90 ± 1.33 months) and group B (6.70 ± 1.12 months) (*P* < 0.05). The postoperative complications such as fractures delayed union and nonunion in group C (2 cases, 8.00%) is less than that in group A (13 cases, 39.39%) and group B (11cases, 37.93%) (*P* < 0.05). The wound infection and needle-tract infection did not differ among the three groups (*P* > 0.05). The excellent or good rate of ankle function was 69.70% in group A, 72.41% in group B and 84.00% in group C, with no statistical difference among the three groups (*P* > 0.05).

**Conclusion:**

Compared with simple external fixator fixation and external fixator combined with plate-screw osteosynthesis, external fixator combined with K-wire intramedullary fixation shortens the operative time and fracture healing time, reduced costs and complications of fracture healing, while the blood loss, infection complications and ankle function recovery showed no difference with the other two groups. External fixator combined with plate-screw osteosynthesis had no advantage in treating extra-articular open fractures of distal tibia and fibula when compared with simple external fixation.

## Background

Open fractures of distal tibia associated with fibula are usually caused by high-energy trauma such as traffic accidents or falling from high places. Till now it is still a big challenge to treat it because of the wound contamination, limited soft tissue envelope and poor vascularity. The role of fibular fixation in the treatment of distal tibiofibular syndesmosis injury and pilon fractures has been well defined [1, 2], however, it is still controversial whether the fracture of fibular needs fixation and which method is selected in extra-articular fractures of the lower leg [3–5].

Bonnevialle [6] conducted a prospective cohort study, 126 cases of patients with distal tibia and fibula fractures were treated by fixing tibia with external fixator, intramedullary nail or plates, while fibula is not fixed, or fixed with intramedullary nail or plates. The results showed that fibula fixation was helpful to restore tibial length, reduce lateral movement of fracture and maintain the stability of the tibia in shaft direction. However, some other studies found that standard open reduction and internal fixation of tibia with intramedullary nail or minimally invasive percutaneous plate osteosynthesis (MIPPO), without the fibula being fixed, had good reduction stability of tibial fracture, reduced the risk of soft tissue injury and infection, and had a good prognosis of function [7–9]. Javdan [10] reported that dynamic compression bone plate (DCP) or tubular plate fixation of fibula had no significant difference in influence on tibial fracture healing, reduction of postoperative complications and recovery of affected limb function. In addition, a few studies have shown that fibular plate internal fixation can increase the rate of delayed healing and non-healing of tibial fractures [1]. In summary, in previous studies on the fixation of fibula fractures was with plate-screw internal fixation, elastic intramedullary nail or non-fixation, no attempt has been made to fix the fibula with Kirschner wires (K-wires).

**Fig. 1:**
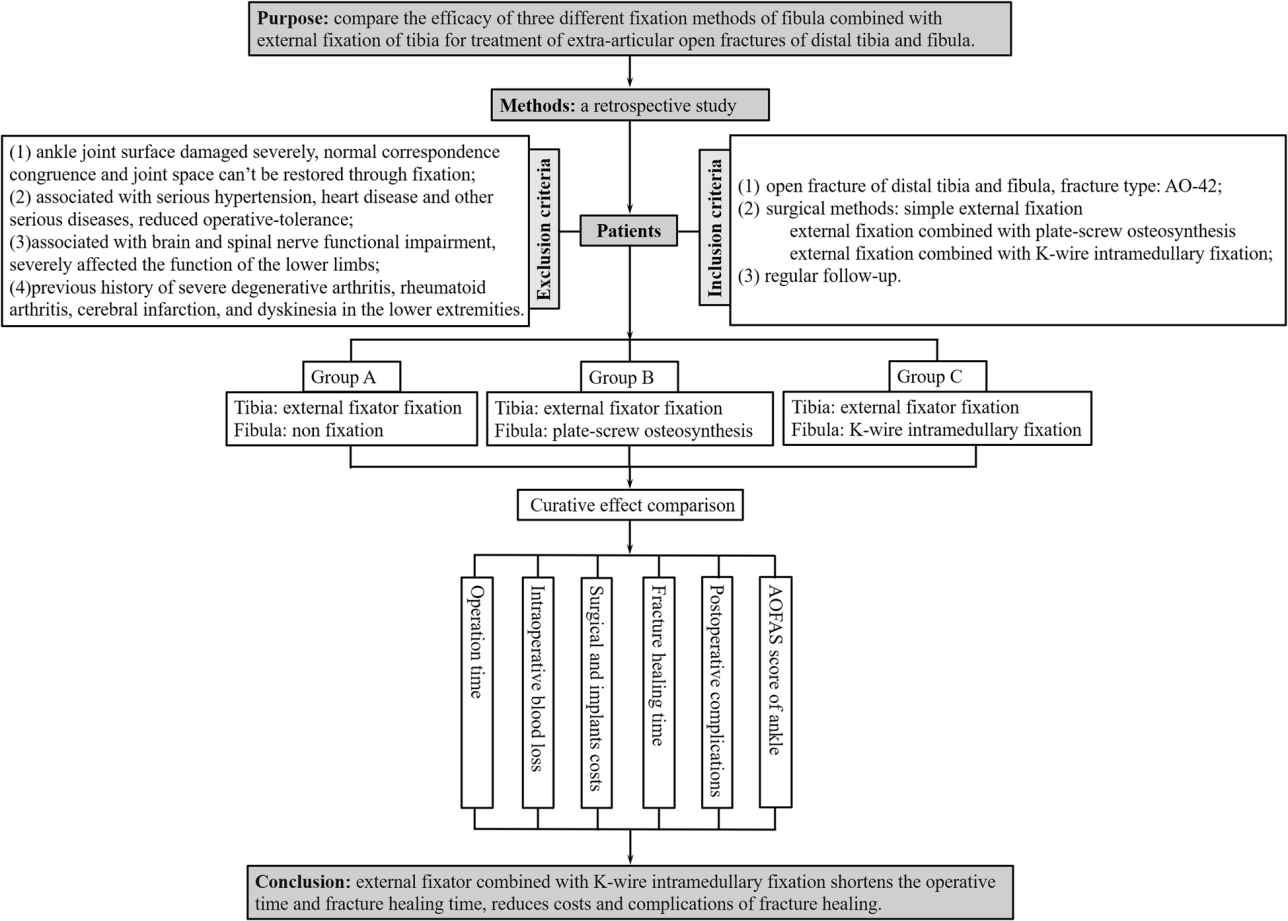
Graphic abstract (AOFAS: American Orthopaedic Foot and Ankle Surgery)

External Fixators always is indicated in open fractures, they are advised in periarticular unstable fractures, floating knee injuries or most commonly in compound fractures in diaphyseal area [11–13]. Besides they are also commonly used always in limited economic reality [14]. The purpose of this study was to compare the efficacy of simple external fixation, external fixation combined with plate-screw osteosynthesis, external fixation combined with K-wire internal fixation for the treatment of extra-articular open fractures of distal tibia and fibula. The operation time, intraoperative blood loss, surgical and implants costs, fracture healing time, postoperative complications, and American Orthopaedic Foot and Ankle Surgery (AOFAS) scores were compared among the groups (Fig. 1).

## Methods

### Materials

External fixators (Hoffmann II®, Wuhan Constant Science and Technology Ltd. Hubei, China), steel plate and screws (Beijing BEST BIO Technical Co., Ltd. Beijing, China), K-wire (Suzhou Gemmed Medical Instrument Co., Ltd. Jiangsu, China), vacuum sealing drainage devices (VSD) (Wuhan VSD Medical Science and Technology Co. Ltd. Hubei, China).

### Patients

The inclusion criteria for this study is as follow: (1) open fracture of distal tibia and fibula, fracture type: AO-42; (2) surgical methods: simple external fixation, external fixation combined with plate-screw osteosynthesis, external fixation combined with K-wire intramedullary fixation; (3) regular follow-up. Exclusion criteria: (1) ankle joint surface damaged severely, normal correspondence congruence and joint space can’t be restored through fixation; (2) associated with serious hypertension, heart disease and other serious diseases, reduced operative-tolerance; (3) associated with brain and spinal nerve functional impairment, severely affected the function of the lower limbs; (4) previous history of severe degenerative arthritis, rheumatoid arthritis, cerebral infarction, and dyskinesia in the lower extremities.

From January 2017 to July 2019, 91 cases of open fractures of distal tibia and fibula were enrolled into this study, among which 35 patients were treated by simple external fixation (group A, *n* = 35), 30 patients were treated by external fixation combined with plate-screw osteosynthesis (group B, *n* = 30), and 26 patients were treated by external fixation combined with K-wire (2.0–3.0 mm) intramedullary fixation (group C, *n* = 26). There was no significant difference in gender, age, cause of injury, Gustilo classification and AO classification among the three groups (*P* > 0.05) (Table 1).

**Table 1:** General information

Group	Patients (n)	Gender		Age (years, x ± s)	Mechanism of injury		Gustilo classification			AO classification		
		Male	Female		Traffic injuries	Others	I	II	III	42-A	42-B	42-C
A	35	27	8	46.83 ± 15.83	25	10	4	12	19	13	16	6
B	30	25	5	44.27 ± 12.37	21	9	7	11	12	11	14	5
C	26	22	4	44.96 ± 14.48	19	7	3	7	16	11	11	4
*P*	–	>0.05		>0.05	>0.05		>0.05			>0.05		

Group A: simple external fixator fixation

Group B: external fixator combined with plate-screw osteosynthesis

Group C: external fixator combined with k-wire intramedullary fixation

### Surgical procedures

#### Emergency debridement

All patients were given emergency debridement. After successful anesthesia, the patient was put in the supine position, plenty of 0.9% saline, hydrogen peroxide and iodophor were used to flushing the wound, disposable negative pressure washing gun could be choose if the wound was deep and polluted seriously. Pneumatic tourniquets are available for operations about the root of patient’s thigh and the lower limb may be prepared and draped before the tourniquet is applied. If necessary, the incision should be prolonged along the wound location to remove contaminated tissue and skin edges, inactivated or suspected inactivated tissue. Investigate the wound carefully and find out whether the fracture end is exposed and if there were vascular and nerve injuries, marked the injured vessels and nerves with silk thread, and reconstructed them after the fracture was fixed. The patients injured in 8 h were fixated according to the general condition after debridement. For patients who were injured over 8 h, calcaneal traction was given temporarily, and fracture fixation should be completed within 7 days.

#### Fracture stabilization

Three groups of patients were treated respectively with simple external fixator (Group A), external fixator combined with plate-screw osteosynthesis (Group B), external fixator combined with K-wire intramedullary fixation (Group C) for fracture reduction and fixation.

**Fig. 2:**
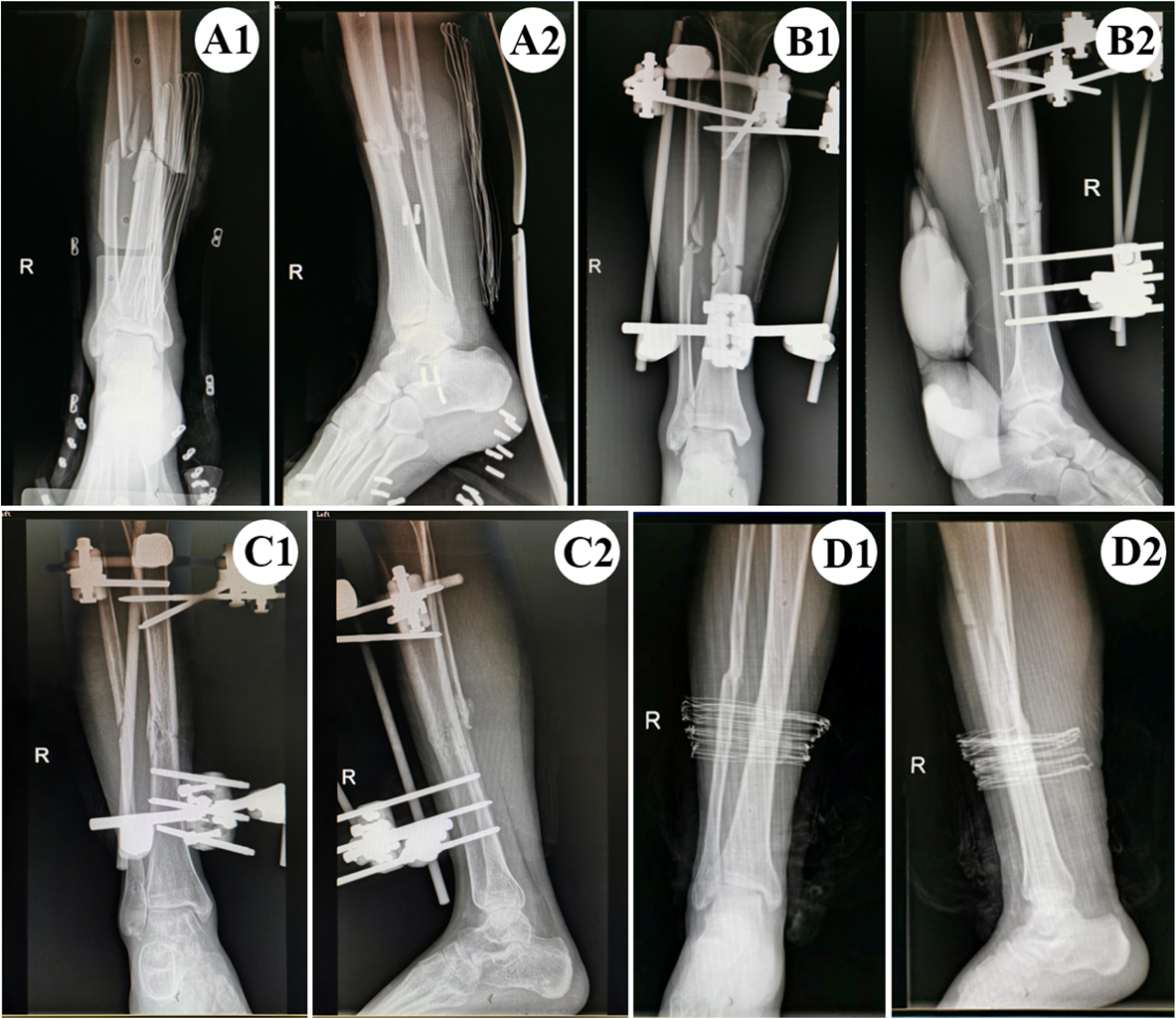
A1-A2 Anteroposterior and lateral radiograph of distal tibiofibular fractures before operation. B1-B2 Bedside X-ray examination 2 days after operation (anteroposterior and lateral views). C1-C2 The fracture showed radiological signs of healing 7 months after operation (anteroposterior and lateral views). D1-D2 The external fixator was removed completely 10 months after operation (anteroposterior and lateral views)

In group A, closed reduction was performed first, fracture ends were antagonized traction and restored with the help of C-arm. If the reduction is not ideal, cut a 3–4 cm incision for exposing fracture ends of tibia and cleaning soft tissue and blood clots embedded in the fracture site. Then the fracture end could be reduced by traction and temporary fixation assisted with bone-holder or K-wire. After the initial reduction of the fracture, inserted screws into the proximal and distal segments of tibia fracture end, the calcaneus or the first metatarsal bone depending on the location of the fracture, installed the fixation clips for each screw and connected them with a biplanar external fixators. The C-arm fluoroscopy was used to examine and adjust the reduction to be in good alignment, then the screw of the fixator was fastened completely. Fracture ends of fibula were not fixed (Fig. 2).

**Fig. 3:**
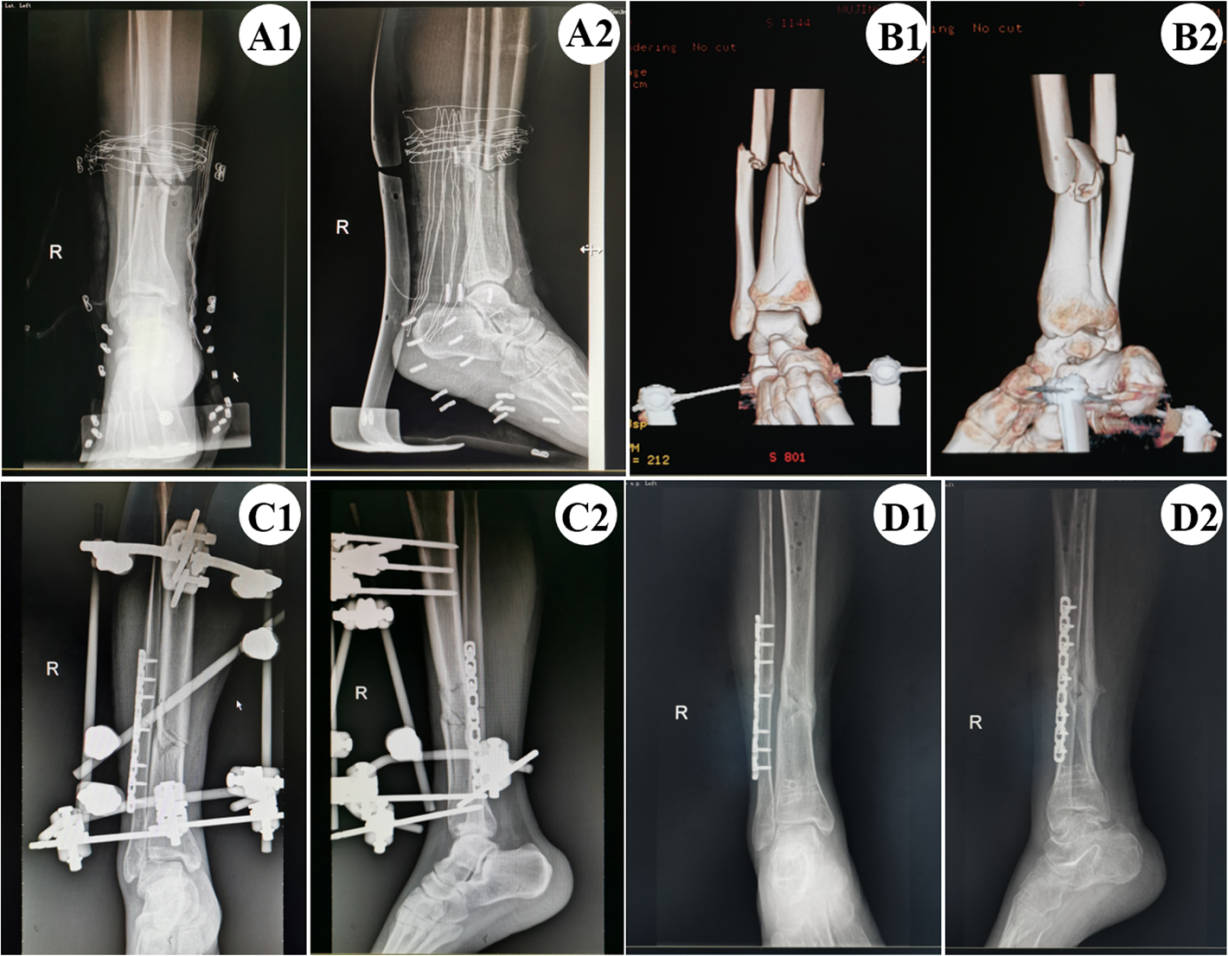
A1-A2 Anteroposterior and lateral X-ray examination of distal tibiofibular fractures before operation. B1-B2 The 3D reconstruction image of CT scan after calcaneal traction. C1-C2 Bedside X-ray radiograph 2 days after operation (anteroposterior and lateral views). D1-D2 The fracture showed radiological signs of healing 6 months after operation and the external fixator was removed completely (anteroposterior and lateral views)

For patients in group B, fracture ends of tibia were fixed with external fixator first in the same way with group A. Then an incision about 10 cm was made centered on the fracture ends of fibula, cut the skin and subcutaneous tissue layer by layer, peeled off the periosteum locally, exposed the fracture ends of fibula, checked the fracture situation, cleaned up the fracture ends and fixed the fracture reduction with bone-holder temporarily, then fixed the fractures with an appropriate length locked plate. The anterior-posterior and lateral views should both be checked and rotary restoration should be confirmed. Once functional reduction is accomplished, the locking screws are driven in (Fig. 3).

**Fig. 4:**
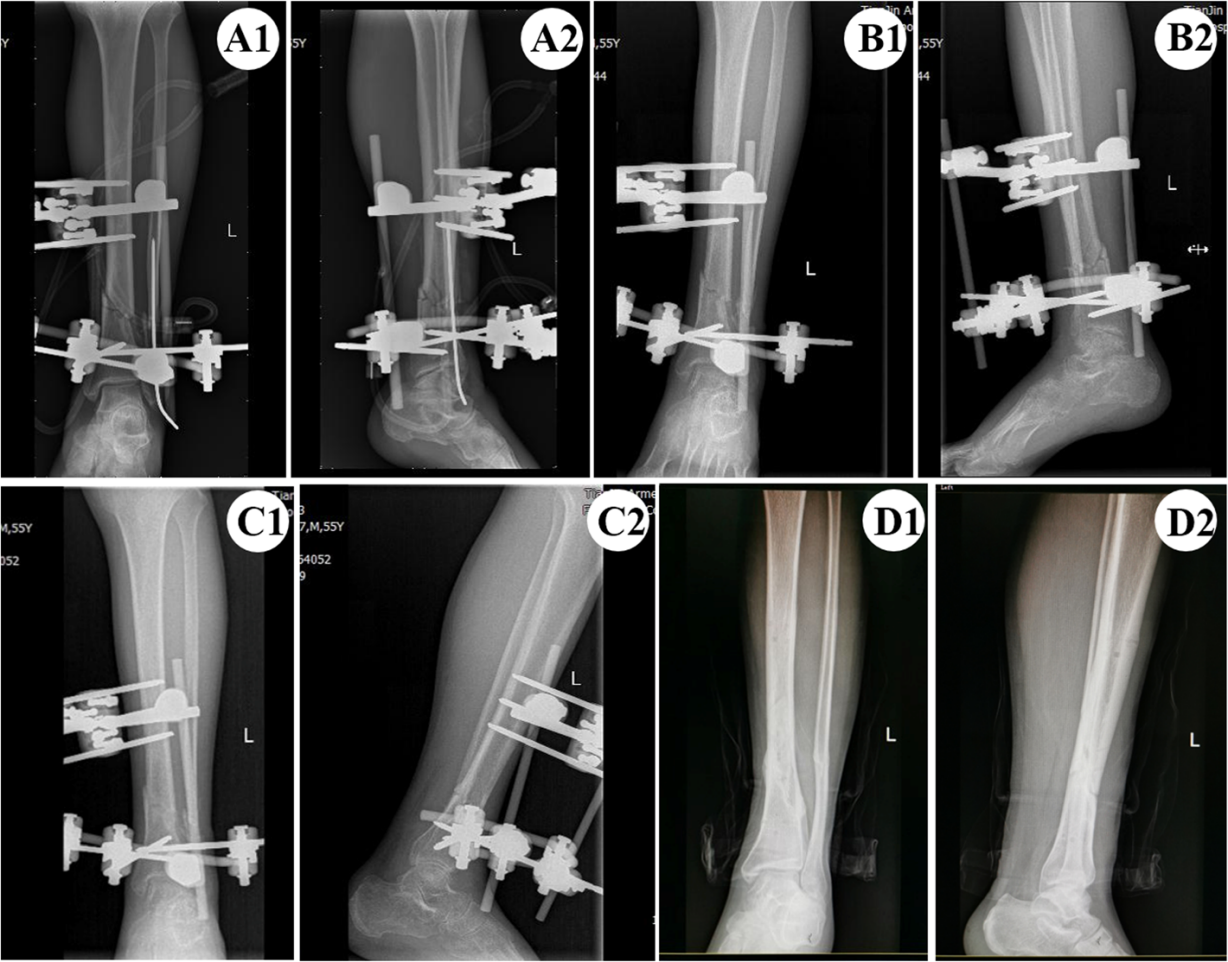
A1-A2 Bedside X-ray radiograph 2 days after operation (anteroposterior and lateral views). B1-B2. The K-wire was removed 8 weeks after operation (anteroposterior and lateral radiograph). C1-C2 The fracture showed radiological signs of healing 5 months after operation (anteroposterior and lateral views). D1-D2 The external fixator was removed completely 8 months after operation (anteroposterior and lateral views)

External fixation combined with K-wire intramedullary fixation was conducted in group C. Firstly, fracture ends of fibula were fixed intramedullary with a K-wire. A 0.5 cm incision was made 2.0 cm above the tip of fibula, and a K-wire of 2.0–3.0 mm diameter was inserted retrograde into the fibular marrow cavity from the bottom to the top. If necessary, a hammer could be used to tap the tail of the Kirschner needle to help insert the needle. During the process of inserting the needle, the K-wire should be parallel to the long axis of the tibia, the multi-segment fractures of fibula were restored in turn with the help of assistant. Adjust the K-wire under the C-arm examination until the fracture of the fibula was in good alignment. The tip of the K-wire should be 10.0 cm more passed through the proximal end of the fibula fracture so as to achieve an effective fixation. The tail of the needle is reflexed outside the skin for easy daily disinfection and care. After fibula fracture was fixed, external fixator was used to fix tibia fracture in the same way with group A (Fig. 4).

#### Treatment of complications

After the fracture was fixed, washing the wound with plenty of saline, then reconstructed the injured blood vessels and nerve. Made primary suture for Gustilo I and Gustilo II patients with less pollution and soft tissue contusion. The patients of type III A, type III B and type III C were treated with partial tension reduction suture because of the large wound and serious pollution. Covered the wound with VSD membranes and connected to a negative pressure drainage tube. For patients with large area of skin and soft tissue defects, Shengjigao (Tianjin Hospital, Tianjin, China) was applied to the wound after 2 weeks from operation to promote rehabilitation. Flap transfer or skin grafting should be performed in the second stage after the improvement of the soft tissue condition of the wound.

#### Postoperative treatment and follow-up

All the patients were given routine antibiotics in the perioperative period to prevent infection, and postoperative analgesia, improving circulation, bone strengthening, prevention of thrombosis and other symptomatic supportive treatment. The wound was treated by dressing change or replacement of VSD regularly and the tail of the K-wire was disinfected with iodophor every day to prevent the infection of wound and pin tract. The patient’s contact information was registered at the time of discharge, and the patient was reviewed periodically. Fracture healing of the tibial and fibula has been observed with X-ray diagnostic techniques. After 8 ~ 12 weeks of follow-up, the K-wire was removed according to the status of external fixation and fracture healing. Later, the patients were followed-up every 3 months and the external fixator was removed when the clinical healing of the fracture was confirmed. During the subsequent visit, professional rehabilitation training guidance should be given according to the different stages of the patients’ recovery. The patient’s limb function was evaluated at the last follow-up.

### Data collection and analysis

The operation time, intraoperative blood loss, surgical and implants costs, fracture healing time, postoperative complications and AOFAS scores at the last follow-up were recorded in the three groups. Fracture healing was assessed by X-ray examination and union was defined as dense callus bridging at least three of four cortices on positive and lateral X-ray examination. Delayed union was defined as radiographic union after > 8 months. Nonunion was defined as lack of any healing within 12 months [15]. Data analysis was performed by SPSS software, version 25.0 (SPSS, Inc., Chicago, IL, USA). Continuous variables were expressed as mean ± SD and differences in continuous variables among groups were examined using analysis of variance (ANOVA) and repeated measure of ANOVA. Pearson’s chi-squared analysis (χ^2^ test) and Fisher’s exact test were used for categorical variables. The level of significance was set at *P* < 0.05.

## Results

In this study, 87 cases were followed up regularly and 4 cases were lost. The follow-up period ranged from 5 to 35 months, with an average of 14.2 months. Four patients who were lost to follow up because they all came from other places. After the operation, the patients returned to the local hospital for further treatment. The results are presented in Table 2.

**Table 2:** Comparison of statistic data for major results after operation among the three groups

Group	Patients (n)	Operation Time(min)	Blood Loss (ml)	Costs* (1000 $)	Imageological healing time of fracture(months)	Complications						Ankle function excellent and good rates(%)
						Fracture union, n			Infection, n			
						Delayed union	Non-union	Total	Wound infection	Pin tract infection	Total	
A	33	142.27 ± 47.05	177.27 ± 134.68	6.48 ± 1.11	6.90 ± 1.33	6	7	13	2	2	4	69.70%(23/33)
B	29	184.00 ± 48.56^a^	206.21 ± 112.48	9.37 ± 2.16^a^	6.70 ± 1.12	7	4	11	4	3	7	72.41%(21/29)
C	25	114.92 ± 36.09^ab^	157.60 ± 72.98	5.24 ± 1.21^ab^	5.67 ± 1.42^ab^	1	1	2^ab^	1	2	3	84.00%(21/25)
P	–	<0.05	>0.05	<0.05	<0.05	–	–	<0.05	>0.05	>0.05	>0.05	>0.05

Group A: simple external fixator fixation

Group B: external fixator combined with plate-screw osteosynthesis

Group C: external fixator combined with k-wire intramedullary fixation

Costs*: include surgical costs and implants costs such as external fixator, steel plate, screws and kirschner wire

^a^*P*<0.05 compared with Group A

^b^*P*<0.05 compared with Group B

## Discussion

Open double fractures of the distal tibia and fibula are complex injuries, if not treated properly, they may cause delayed union or nonunion of fractures, severe cases may lead to traumatic arthritis, which seriously affects the function of ankle joint. In 2015, Professor Raman Mundi et al. [3] published an update of the treatment guidelines for open tibial fractures in JBJS REV magazine. At present, intramedullary nail is recommended for open tibial shaft fractures, but the incidence of ankle stiffness, residual ankle pain and traumatic arthritis after intramedullary nail fixation is high. In addition, intramedullary nail has poor fixation effect on distal tibial comminuted fractures. Compared with the intramedullary nail, composite external fixator is flexible in assembly, simple in operation, short in operation time, and less in intraoperative blood loss. The external fixator can perform compression fixation, stretch fixation and neutral fixation according to different types of tibial-fibular fractures. It is a limited elastic fixation, which reduces the stress shielding of static locking fixation, and is beneficial for the fretting of the longitudinal axis of the shaft at the same time. As a result, it can promote the healing of the fracture by making the fracture end get physiological stress.

However, pin tract infections are the big problem which is commonly occur in children with additional trauma and the people lack of selfcare [16]. So, external fixator is traditionally used as temporary fixator for open tibia and fibula fracture, which requires second-stage operation to be converted to internal fixation [17, 18]. In recent years, many studies have shown that external fixator can also be used for definitive fixation of open tibia and fibula fractures [19–22]. Some research reported that external fixation showed similar results to intramedullary nailing for treatment of open fractures of open tibia and fibula in terms of infection rate, malalignment, bone healing and quality of life [23–25]. The other researchers found that compared with simple external fixation, external fixation combined with limited internal fixation is an effective and safe alternative for management of open tibial diaphyseal fractures. It provides superior initial reduction, better stability and decreases the risk of inferior alignment and delayed union without increasing the risk of infection [26–28].

Recently, the role of fibula has been paid more attention by surgeons. Research shows that fibula bears 6.4% of body weight in ankle neutral position and the number can be more in ankle dorsiflexion or valgus. The stress distribution of medial tibia is larger than that of lateral tibia under normal physiological load, however when the fibula loses its continuity, the stress distribution will concentrate on the outside of the tibia [7]. The change of the stress distribution of the tibia will make the negative gravity line move out and cause ankle joint disorder. Over-syndesmotic treatment of the fibula fracture can make tibia osteosynthesis more efficient and improve the outcome [29]. Therefore, the fixation of fibula is according with the principle of biomechanics [30].

Through the comparative study of three different fixation methods of fibula combined with tibia external fixation, it was found that external fixator combined with plate-screw osteosynthesis in the treatment of open fracture of distal tibia and fibula prolonged the operation time, but had no obvious advantages in promoting fracture healing, reducing postoperative complications and functional recovery of affected limbs. The good news is that the K-wire group shortened the operation time and fracture healing time, reduced the costs and complications of fracture healing when compared with the other two groups. However, there was no significant difference among the three groups in terms of intraoperative blood loss, postoperative infection complications and the score of ankle function. The main reason led to this situation may be that the open fractures of distal tibia and fibula was often caused by high-energy trauma, which usually accompanied with other partial fractures and severe soft tissue contusions.

K-wire was first introduced as an internal fixation implant in 1909 by Martin Kirschner and it had been used for more than 110 years in orthopedic surgery [31]. Today K-wire is commonly used as a kind of temporary fixation device in clinic, however it can also been used as definitive fixator in displaced metacarpal shaft fractures, clavicle fractures and paediatric tibial shaft fractures [32–34]. The bone marrow cavity of fibula is narrow and irregular in shape, so the K-wire can be used to fix fibula fracture according to the principle of multi-point fixation [35]. Its advantages are as follows: first, no reamed medulla cavity, no peeling of periosteum, no destruction of the original hematoma at the fracture end, no aggravation of secondary damage to the skin, soft tissue and blood supply at the fracture site. It reduces complications of skin and soft tissue effectively and reflects the concept of minimally invasive treatment of fractures. Secondly, K-wire intramedullary fixation is easy to operate and does not require special equipment, it can be removed out at clinic without hospitalization. Thirdly, K-wire had lower economic cost but higher cost-effectiveness ratio when compared with locked plate and elastic intramedullary nail. Fibular K-wire intramedullary fixation conforms to the concept of biological fixation (BO) [36], whose core idea was to protect the blood supply of the fracture end, it didn’t emphasize anatomical reduction, but to seek a balance between the stability of the fracture and the blood supply of local soft tissue. This fixation method belongs to elastic fixation, which didn’t require very strong fixation, but can reduce the stress shielding of tibial healing. In addition, micromotion in elastic fixation of fracture ends can promote revascularization, rapid calcification of callus and the formation of related osteogenic factors, thus promoting the healing of fibular fractures. External fixator combined with K-wire had an excellent fixation effect in fixing comminuted distal tibial fracture. It can restore the continuity of fibula by K-wire and reduce the weight of tibia. Percutaneous k-wire fixation is a minimally invasive method and far away from the fracture end, thus it can maximize the protection of periosteum and soft tissue blood supply at the fracture site, and avoid the destruction of intramedullary nail fixation on the blood supply of the medullary cavity, which conforms to the concept of damage control orthopedics (DCO) [37]. More important, combined fixation increased the stability of tibial fixation, reduced the excessive stress damage of fracture end, and improved the healing rate of tibia fracture.

In conclusion, the importance of fibular fixation in the treatment of open fracture of distal third tibia and fibula has been accepted by more and more surgeons, but the method of fixation has not reached a consensus. Proper fibular internal fixation can promote the healing of tibial fracture and maintain the stability of lower tibiofibular joint and ankle joint. Compared with simple external fixation and external fixation combined with plate-screw osteosynthesis, external fixation combined with K-wire has less trauma, shorter operation time, lower costs, faster fracture healing and better healing quality of fracture in the treatment of open fracture of distal tibia and fibula. This study also has some limitations, it is a retrospective case study with small sample size, lack of prospective cohort study and randomized controlled study. The results need to be further confirmed by large sample clinical studies.

## Conclusion

Compared with simple external fixator fixation and external fixator combined with plate-screw osteosynthesis, external fixator combined with K-wire intramedullary fixation shorten the operative time and fracture healing time, reduced costs and complications of fracture healing, while the blood loss, infection complications and ankle function recovery showed no difference with the other two groups. External fixator combined with plate-screw osteosynthesis had no advantage in treating extra-articular open fractures of distal tibia and fibula when compared with simple external fixation.

## Data Availability

Not applicable.

## Abbreviations

K-wire: Kirschner wire
AOFAS: American Orthopaedic Foot and Ankle surgery
DCP: Dynamic compression bone plate
MIPPO: Minimally invasive percutaneous plate osteosynthesis
VSD: Vacuum sealing drainage devices
ANOVA: Analysis of variance
BO: Biological fixation
DCO: Damage control orthopedics
